# To the Editors of the Medical and Physical Journal

**Published:** 1806-03

**Authors:** Thomas Fitzpatrick

**Affiliations:** Trim, County Meath


### *To the Editors of the Medical and Physical Journal.*

GENTLEMEN,

THE following Case appearing to me as a rare occurrence, induces me to offer it for your perusal.

Judith Commons, aged 23 years, of delicate form and scrofulous appearance. About two months subsequent to the birth of her first child, which was attended with no untoward circumstance, perceived a tumor in each groin, exciting considerable pain, which was alleviated, by their opening spontaneously, and discharging a quantity of matter, at first purulent, but gradually becoming ichorous; the edges of the ulcers assuming a bluish hue. She now became apprehensive that her illness was syphilitic, and in this, was supported by a concurring medical opinion. Her husband, convinced of his never having had any species of the disease, seeing the child perfectly healthy, and relying on her chastity, requested my advice. On my first visit, which was made the sixth week of her indisposition, I found the abdomen much enlarged, but without any evident fluctuation; legs œdematous; urine scanty and high coloured; appetite much impaired; ulcers very superficial and stationary. Doubtful as to the nature of her disease, and timid in giving an opinion, different from that of a man much longer in the profession, I declined offering my sentiments, but proposed the administration of such medicines as might tend to alleviate her present symptoms. Mercurial preparations presented themselves as most deserving a trial; they were had recourse to, in form of friction; pills composed of calomel, squill and digitalis were directed, and the ulcers to be dressed with common cerate. After persevering in this treatment for five weeks, the dropsical symptoms were entirely removed, and the sores nearly healed; her appetite returned, and she seemed in a great measure restored to her wonted strength. Confined circumstances, however, deprived her of that nutritious diet, and suitable exercise, so necessary to restore tone and energy to a system exhausted by tedious illness.

A few weeks only elapsed, when most of the symptoms, already enumerated, recurred with increased violence. The abdomen became again distended, the umbilicus prominent and much inflamed; and through the integuments the mesenteric glands could be distinctly felt, much enlarged, and seemingly indurated. She was now, by her own own desire, removed into the country, and bought a supply of the medicines above advised, (with the addition of extractum cicutæ to the pills) cloths wet in a saturnine solution were applied to the umbilicus with the intention of removing the inflammation.

From this period, August 10, until the 6th of November, I had no opportunity of seeing her; when it appeared in vain to attempt resisting her dissolution. Conjoined with those symptoms above mentioned, there was an opening nearly two inches in circumference, and about three in a direct line above the umbilicus, which seemingly communicated with the colon; through this, fæces constantly oozed, though sometimes passed per anum. Under those circumstances she had existed five weeks, and frequently expressed a desire for food. She had constant evening exacerbations; pulse scarcely perceptible; countenance expressive of extreme agony; urine small in quantity, but not passed involuntarily; her mental faculties remained in a great degree unimpaired, until the last moments of her existence. From the obstinacy of her friends, I could not be permitted to inspect the body after death.

I am, &c.

*Trim, County Meath,* THOMAS FITZPATRICK, M. D.

*Jan.* 19, 1806.

P. S. I should have observed, that the sores in the groin were compleatly cicatrized.

